# Gold

**Published:** 1895-01-12

**Authors:** 


### The Panacea in English Medicine.

### II.—GOLD.

All ancient authorities on medicine concurred in assigning great efficacy to gold. It was held to have special virtues in strengthening the heart, and hence Chaucer's sly allusion in his description of the Doctor of Physike:—

> He kepte that he wan in pestilence.
> For gold in phisik is a cordial;
> Therefore he lovede gold in special.

The difficulty of reducing gold to a form capable of being assimilated by the patient's system was, however, an insuperable bar to its common use. Unless dissolved by powerful corrosives, rendering it dangerous and hurtful in the extreme, it remained practically unaffected by the most complicated processes, and resisted every effort to reduce it to a liquid form. Nevertheless, it continued an important ingredient in the many Waters of Health which abounded in Elizabethan days, and was prescribed by the regular physicians, partly out of respect to old authorities and partly, it may be suspected, from some lurking belief in its occult powers.

It remained for Francis Anthonie (b. 1550, d. 1623) to claim the honour of first reducing gold to a potable form, with the inevitable consequence of exalting it to the position of a Catholicon, or universal medicine. The father of Anthonie was a goldsmith of considerable reputation, and his early associations no doubt contributed to set him on the track of the researches to which he dedicated his life. He was educated at Cambridge, and continued his studies at the University till middle life. In 1598 he published a little Latin pamphlet at Hamburg, "Panacea Aurea," on the subject which had begun to engross him, and two years later, encouraged by the success of his theories abroad, he came up to London, and without going through any preliminary study of medicine, began to practise indiscriminately with his new preparation. Those were not days when any presumption or novelty could pass unrebuked. Anthonie was promptly summoned before the Royal College of Physicians, and charged with administering without licence "diaphoretick medicines prepared from gold and mercury." His doses of the latter drug must have been tolerably bold, since the evidence showed that one of his patients had dropped all his teeth. Anthonie was forced to undergo the usual examination in medicine before a select board, was found "very weak and ignorant" in the rudiments of his adopted profession, and was condemned to pay a fine of £5, "propter illicitam Praxin." Refusing to pay this fine, Anthonie was released from the debtors' prison by the order of the Lord Chief Justice; but, greatly incensed at the breach of their privileges, the College appealed, and eventually forced Anthonie to submit. The quarrel did not end there, for Anthonie continued to administer his "Aurum Potabile," and firmly refused to pay the accumulated fines imposed by the College. He was lodged for many months in the debtors' prison, then an unspeakably foul and dangerous quarter, and was only released at the last owing to the personal intercession of his wife with the College, aided probably by the force of public opinion, for Anthonie was now becoming a very famous personage. The degree of Doctor of Physic was conferred upon him by the Universities, and among his patients were some of the most distinguished men of the day. As in the case of all empirics, many notable cures in small-pox, plague, and other virulent diseases are recorded of him. And what is more remarkable, he succeeded in imposing his theories on many scientific inquirers of his own and the succeeding age.

A few years later he published, first in Latin and then in English, a justification of his "Aurum Potabile," in which he is concerned to prove first that his drug is a universal medicine, and secondly that it is actually what it professes to be, gold in solution. He is forced to except from the operation of his panacea many incurable diseases, chiefly of an hereditary character. He argues that since certain medicines are applicable to many diseases, and hence are known as Polychresta, it is agreeable to reason that one drug should be found applicable to all diseases, and for this theory he relies much on the authority of Raymond, Lully, and Arnaldus. It is a curious mark of the times that the principal argument used against him by his opponents was not the fallacy of his theories but the defects of his instrument. They vehemently maintained that the preparation sold by him at the rate of 5s. an ounce, under the name of "Aurum Potabile," contained no trace of gold whatever, and the "Aurum non Aurum" controversy raged hotly during the remaining years of Dr. Anthonie's life. As the secret of its preparation was very jealously guarded by its author, he was unable to justify himself further than by pointing out that his elixir produced the effects universally ascribed to "Aurum Potabile," and must on this account be admitted to be genuine. His most bitter opponent was a certain Dr. Cotta, a Nottingham physician, some of whose patients had fallen under the influence of Anthonie and his gold cure. Cotta wrote in 1614 what has been called "a passionate and waspish" treatise, to prove that gold could be dissolved in no other way than by the use of corrosives. He was persuaded by Anthonie's influential friends to withhold the attack from publication, but on some fresh provocation arising he gave it to the press under the title of "Cotta contra Antonium" in 1623, a few months only before Anthonie's death.

The great secret of the preparation transpired many years after. The famous menstruum, or dissolving medium, was formed from a mixture of "philosophic vinegar" (distilled from six gallons to ten quarts) and "philosophic salt" made from calcined block tin. The gold was to be filed into small dust, melted in a crucible with white salt, and ground on a painter's stone. Being then purified from the salt a very fine white calx remained. It was to be mixed with half a pint of the menstruum and distilled again and again. A clammy substance of the consistency of honey was left behind, one ounce of which put into a quart of pure canary wine was the "Aurum Potabile."

It must be left to the reader to decide whether Dr. Cotta and his "Aurum non Aurum" partisans had not some justification for their scepticism.

